# Computer-Aided Lung Nodule Recognition by SVM Classifier Based on Combination of Random Undersampling and SMOTE

**DOI:** 10.1155/2015/368674

**Published:** 2015-04-06

**Authors:** Yuan Sui, Ying Wei, Dazhe Zhao

**Affiliations:** ^1^Software College, Northeastern University, Shenyang 110004, China; ^2^School of Information Science and Engineering, Northeastern University, Shenyang 110004, China; ^3^Key Laboratory of Medical Imaging Calculation of the Ministry of Education, Shenyang 110004, China

## Abstract

In lung cancer computer-aided detection/diagnosis (CAD) systems, classification of regions of interest (ROI) is often used to detect/diagnose lung nodule accurately. However, problems of unbalanced datasets often have detrimental effects on the performance of classification. In this paper, both minority and majority classes are resampled to increase the generalization ability. We propose a novel SVM classifier combined with random undersampling (RU) and SMOTE for lung nodule recognition. The combinations of the two resampling methods not only achieve a balanced training samples but also remove noise and duplicate information in the training sample and retain useful information to improve the effective data utilization, hence improving performance of SVM algorithm for pulmonary nodules classification under the unbalanced data. Eight features including 2D and 3D features are extracted for training and classification. Experimental results show that for different sizes of training datasets our RU-SMOTE-SVM classifier gets the highest classification accuracy among the four kinds of classifiers, and the average classification accuracy is more than 92.94%.

## 1. Introduction

Nowadays lung cancer is one of the most serious cancers in the world. In fact, the total number of deaths caused by lung cancer is greater than the sum of breast cancer, prostate cancer, and colorectal cancer [1, 2]. Early detection and treatment of lung cancer can improve the survival rate of those inflicted with it [3]. Pulmonary nodules are early manifestations of lung cancer. Lung nodule refers to lung tissue abnormalities that are roughly spherical with round opacity and a diameter of up to 30 mm.

Computed tomography (CT) is an important tool for early detection of nodules, but interpreting the large amount of thoracic CT images is a very challenging task for radiologists. Currently, nodules are mainly detected by one or multiple expert radiologists inspecting CT images of lung. Recent research, however, shows that there may exist interreader variability in the detection of nodules by expert radiologists [4]. An automated system can thus provide initial nodule detection which may help expert radiologists in their decision-making. Computer-aided detection/diagnosis (CAD) is considered a promising tool to aid the radiologist in lung nodule CT interpretation.

In lung cancer CAD systems, lung nodule detection methods can be categorized into three main categories [5]: template-based [6–8], segmentation-based [9–11], and classification-based [12–15]. Among the reported existing work, the systems that included a classification component in their structure have performed better than their counterparts. There is a host of classification algorithms that could be employed to enhance the accuracy of the lung nodule detection. This work is concerned with classification-based lung nodule detection.

However, lung nodule classification is a typical unbalanced dataset problem; that is, the number of nonnodule samples for training is greatly more than that of nodules. For unbalanced datasets, the number of samples in majority class outnumbers the number of samples in the minority class. Rare individuals are typically harder to identify than common objects, and most machine learning algorithms have many difficulties in dealing with rarity; it is important to study the classification problem of unbalance dataset.

Support vector machine (SVM) is a new machine learning method based on statistical learning theory [16]. It overcomes many shortcomings such as over learning, the local extreme points, and dimensionality disaster that the neural network and traditional classifiers have. SVM has strong generalization ability and has now become a new hotspot in the field of machine learning. However, in a conventional SVM classifier, a highly unbalanced distribution of data usually brings about poor classification accuracy for the minority class, because the classifier may be strongly biased toward the majority class. SVMs tend to learn how to predict the majority class in particular, although they can get higher predictive accuracies without considering the minority class; this good performance can be identified as meaningless.

In recent years, the machine learning community has addressed the issue of class imbalance mainly in two different ways [17–19]. The first way involves modifying the classifiers or putting forward new algorithms to adapt to the unbalanced datasets [20]. The second classifier independent way involves balancing the original data set, for example, oversampling [21, 22] and undersampling [23, 24]. Chawla et al. [25] proposed the synthetic minority oversampling technique (SMOTE) algorithm in which the minority class was oversampled by taking each minority class sample and introducing new synthetic examples joining any or all of the minority class nearest neighbors. Some used a combination of undersampling and oversampling, such as Estabrooks et al. [26], who concluded that combining different expressions of resampling approach was an effective solution. Researchers then exerted their efforts toward developing hybrid approaches to deal with unbalanced data, where they combined oversampling and undersampling with different concepts into one approach.

For SVM classifier, the key issue to improve the performance of SVM classification under unbalanced dataset is how to ensure that the data become balanced, and at the same time, utilizing the sample information to generate more effective decision-making interface.

From the above analysis, in order to improve SVM algorithm's classification performance under unbalanced dataset for lung nodules detection, we propose a SVM classification algorithm based on random undersampling and synthetic minority oversample technique (SMOTE). The combination of the two methods not only achieves balanced training samples, but also removes noise and duplicate information in the training sample and retains useful information to improve the effectiveness of data utilization and ultimately improves performance of SVM algorithm for pulmonary nodules classification under the unbalanced data. The rest of the paper is organized as follows.  Section 2 analyses conventional SVM and effect of unbalanced dataset for the performance of classification, explains the architecture of the proposed balancing approach, and presents a description of the dataset and the experimental method used in this research. Results and discussions are presented in Section 3.  Section 4 concludes the paper. The features of lung nodule used for classification are introduced in Appendix A.

## 2. Materials and Methods

### 2.1. Conventional SVM and Unbalanced Dataset Problem

#### 2.1.1. Overview of Conventional Support Vector Machine

SVM is a learning procedure based on the statistical learning theory [27, 28] and it is one of the best machine learning techniques used in data mining [29]. For solving a two-class classification problem, the main objective of SVM is to find an optimal separating hyperplane that correctly classifies data points as much as possible and separates the points of the two classes as far as possible by minimizing the risk of misclassifying the training samples and unseen test samples [27].

In the problem of two class pattern recognition, suppose that there are *N* sample points in the training set *s* = {(*x*_*i*_, *y*_*i*_)}, among them *x*_*i*_ ∈ *R* ^*d*^, and *y*_*i*_ ∈ {+1, −1}, *i* = 1,2,…, *N*. SVM is to find the optimal solution of the following quadratic programming problem:

(1) min⁡w,b,ξ 12 wTw+C∑i=1Nξi s.t.  yiw·Φxi+b≥1−ξi, i=1,2,…,N   ξi≥0, i=1,2,…,N,

where *ξ* is slack variable, which indicates the severity of misclassified samples; *C* is a regularization constant, namely, penalty factor, which is used to control the degree of punishment for misclassified samples. In order to derive the dual problem from formula (1), Lagrange function is introduced as follows:

(2) Lw,b,ξ,α,β=12w2+C∑i=1Nξi −∑i=1Nαiyiw·Φxi+b−1+ξi −∑i=1Nβiξi.

Among formula (2), *α*_*i*_ and *β*_*i*_ are Lagrange parameters. Thus the dual problem of formula (1) can be drawn, namely, the following convex quadratic programming problem:

(3) min⁡α 12∑i=1N∑ j=1NαiαjyiyjKxi,xj−∑i=1Nαis.t. ∑i=1Nyiαi=0  0≤αi≤C, i=1,2,…,N.

Formula (3) is the commonly used standard C-SVM model, due to the fact that the calculation of inner product between vectors in a high dimensional space is very difficult and sometimes even impossible. In formula (3), *K*(*x*_*i*_, *x*_*j*_) = Φ(*x*_*i*_) · Φ(*x*_*j*_) is taken with a semipositive definite kernel, which instead of high dimensional vector inner product calculation, and this is kernel trick of SVM.

By solving formula (3), Lagrange parameters can be solved as follows: *α* ^∗^ = (*α*_1_^∗^, *α*_2_^∗^,…,*α*_*N*_^∗^)^*T*^, part of the samples corresponding to *α*_*i*_, whose value is not zero, called support vector. Select *α*_*i*_ that is located in the open interval (0, *C*) to calculate *b* ^∗^ = *y*_*i*_ − (∑_*i*=1_^*N*^ *y*_*i*_ *a*_*i*_^∗^ *K*(*x*_*i*_, *x*_*j*_)) and finally construct the following decision function: *f*(*x*) = sgn(∑_*i*=1_^*n*^ *α*_*i*_^∗^ *y*_*i*_ *K*(*x*, *x*_*i*_) + *b* ^∗^) as the classification rule.

#### 2.1.2. Effect of Unbalanced Data to the Classification Performance of SVM

When the sample sizes of different classes are equivalent or even the same in the dataset, the classification boundary of the SVM classifier is desirable. While, when the sample sizes are different greatly between the two classes, SVMs will run into difficulties [29, 30]. It can be shown from formula (1) that minimizing the first term (1/2)*w* ^*T*^ *w* is equivalent to maximizing the margin, while minimizing the second term ∑*ξ*_*i*_ means minimizing the associated error. The constant parameter *C* is the trade-off between maximizing the margin and minimizing the error. If *C* is not very large, SVM simply learns to classify everything as negative because that makes the margin the largest, with zero cumulative errors on the abundant negative examples [26]. The corresponding trade-off is only the small amount of cumulative error on the positive examples, which do not count for much. Thus, SVM fails in situations with a high degree of unbalance. Besides, SVM tends to produce an insignificant model by almost predicting the majority class; thus the classification result is obviously not desired.

**Figure 1:**
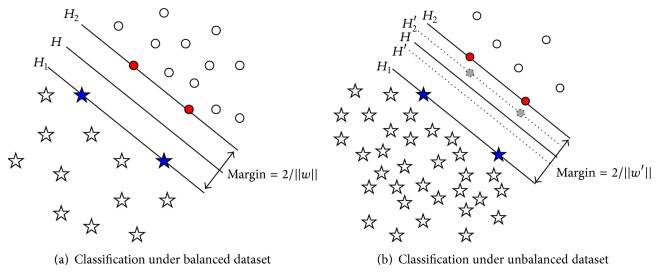
Illustration of SVM classification performance under different datasets.

So the unbalanced dataset will impact the classification performance of SVM. We use an illustration to show the misclassification in Figure 1.

In Figure 1, “circle” indicates minority class sample, and “pentagon” indicates majority class sample. When the number of two class samples is equivalent or balanced as Figure 1(a), “blue pentagons” determine the support vector *H*_1_ of majority class, and “red circles” determine the minority class hyperplane *H*_2_, and the optimal classification hyperplane *H* can be calculated correctly. When the number of two class samples is unbalanced as Figure 1(b), due to the fact that the samples of minority class are rare, some minority class samples which should determine the hyperplane *H*_2_′ did not present, such as “gray circles” on dotted line *H*_2_′. If the Boundary Samples were provided, the calculated classification hyperplanes should be *H*′, *H*_2_′, and *H*_1_, but now the results are *H*, *H*_2_, and *H*_1_; they are apparently different from the truth, so the deviation appears. Actually, the more minority class samples are, the more the calculated results will be close to the truth classification hyperplanes because of the unbalanced samples, which make the majority class hyperplane “push” towards the minority class direction, thus affecting the accuracy of the calculation.

#### 2.1.3. Biased-SVM Model for Unbalanced Samples

As analysed above, for a standard C-SVM model, unbalanced dataset may cause deflective classification results. An effective way to solve the problem is selecting different penalty parameters on two kinds of samples in the SVM model, using larger value of *C* representing more importance for the minority class samples, and taking more strict classification error punishment, which is the basic idea of biased-SVM [31].

In biased-SVM model [31], select different penalty parameters *C*_+_ and *C*_−_ for the two class samples, respectively, so the model can be expressed as:

(4) min⁡w,b,ξ 12 wTw+C+∑i ∣ yi=+1Nξi+C−∑i ∣ yi=−1Nξis.t. yiw·Φxi+b≥1−ξi, i=1,2,…,N  ξi≥0, i=1,2,…,N.

To solve the quadratic programming problem of formula (4), the dual problem is derived by introducing Lagrange factors, and kernel function is also used to avoid high dimension vector dot product. So the model of biased-SVM can be deduced as

(5) min⁡α 12∑i=1N∑ j=1NαiαjyiyjKxi,xj−∑i=1Nαis.t.  ∑i=1Nyiαi=0   0≤αi≤C+, yi=+1   0≤αi≤C−, yi=−1.

### 2.2. Proposed Approach

The intuition of our approach is to balance the samples from two aspects. For the minority class, we apply SMOTE algorithm to create new synthetic examples, without adding too much noise into the dataset; the minority samples will be oversampled. On the other hand, we decrease the redundancy samples of majority class with the remaining of its cluster. Therefore, we combine two resampling techniques of upsampling of minority class and undersampling majority class.

#### 2.2.1. Using SMOTE Algorithm on the Samples of Minority Class

The synthetic minority oversample technique (SMOTE) algorithm proposed by Farquad and Bose [28] is a powerful method for upsampling technique, and it has a very successful performance in different application areas. SMOTE oversampling technology is different from traditional oversampling methods by simple sample-copy. It uses samples of minority class to control the generation and distribution of artificial samples to achieve the purpose of balancing datasets, and it can effectively solve the overfitting problem leading by a narrow decision-making range.

SMOTE algorithm utilizes the similarity of the feature space in the existing samples of the minority class to establish new data. For a subset *S*_min⁡_ ⊂ *S*, its each sample *x*_*i*_ ∈ *S*_min⁡_ uses *K*-nearest neighbor algorithm; *K* is an appointed integer. Here *K*-nearest neighbors are defined as *K* elements whose Euclidean distance to *x*_*i*_ in *n*-dimensional feature space *X* is the minimum values. In order to construct a synthetic sample, first randomly select a *K*-neighbor and then multiply it by the difference with the corresponding eigenvectors and random number among [0,1]. Thus any synthetic instance *x*_*s*_ is given by

(6) $$\begin{array}{c} x_{s}=x_{i}+\delta \cdot \left(x_{i}^{\left(t\right)}-x_{i}\right), \end{array}$$

where *x*_*s*_ denotes one synthetic instance; *x*_*i*_^(*t*)^ is the *t*th nearest neighbors of *x*_*i*_ in the positive (minority) class, and *δ* ∈ [0,1] is a random number. The procedure is repeated for all the minority data points.

**Figure 2:**
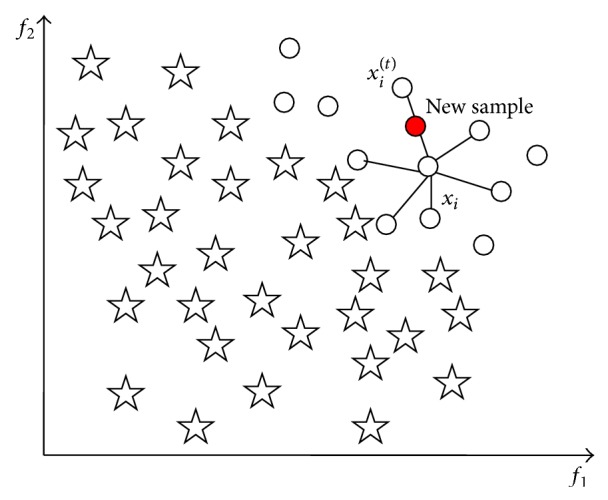
Sample *x*_*i*_, its *K*-nearest neighbors (*K* = 6), and the new synthetic sample by SMOTE.

Figure 2 shows an example of the process of SMOTE, in which there is a typical unbalanced data distribution, and among them circles and pentagons denote samples of minority class and majority class, respectively. In the *K*-nearest neighbors *K* = 6. Figure 1 shows the constructed new sample along the connection-line of *x*_*i*_ and *x*_*i*_^(*t*)^, the newly generated sample using a red solid circle to indicate it clearly. SMOTE algorithm is based on the assumption that a sample constructed between the nearby samples in the minority class is still a sample of minority class. The basic idea of SMOTE algorithm is to get synthetic samples of minority class by oversampling at the connection between the current samples of minority class. For each sample in the minority class, look for the *K*-nearest neighbors at its similar samples and then randomly select one of the *K*-nearest neighbors and construct a new artificial minority class sample between the two samples by linear interpolation method. After SMOTE processing, the number of minority class will increase *K* times. If more artificial minority class samples are needed, repeat the above interpolation process to achieve a balance in the new generated training samples and finally use the new sample dataset for training the classifier.

These synthetic samples help to break the drawback of simple upsampling; the increasing of the original dataset in this way can make the learning capacity of the classifier improve significantly.

#### 2.2.2. Random Undersampling (RU) Algorithm

Unbalanced dataset due to the much more number of majority class samples than that of minority class, as analysed in Section 2.1.2, will seriously affect the performance of SVM. To get a balanced dataset between the two classes, we adopt random undersampling (RU) algorithm to decrease samples of the majority class.

Before random undersampling, suspected noise samples on the boundary of majority class are detected and removed in our method. As shown in Figure 1, the support vector machines and classification hyperplane are mainly determined by those junction samples between two classes, so boundary noise samples of majority class will make the classification hyperplane “invasion” to the minority class direction; thus the classification performance will apparently get worse. In this paper, boundary noise samples of majority class are identified and removed to make the classification more accurate.

Set *x*_maj_, *x*_min⁡_ indicates coordinates of majority class and minority class sample, respectively; *n*_maj_, *n*_min⁡_ are number of majority class and minority class samples; *x*_center_maj_, *x*_center_min⁡_ are centers of the two class samples; *r*_ave_maj_, *r*_ave_min⁡_ are average radius of the two class samples, and they can be calculated as follows:

(7) $$\begin{array}{c} x_{\text{center}\_\text{maj}}=\frac{\sum_{n_{\text{maj}}}x_{\text{maj}}}{n_{\text{maj}}},\;\;x_{\text{center}\_\min}=\frac{\sum_{n_{\min}}x_{\min}}{n_{\min}},\\ r_{\text{ave}\_\text{maj}}=\frac{\sum_{n_{\text{maj}}}\left\|x_{\text{maj}}-x_{\text{center}\_\text{maj}}\right\|}{n_{\text{maj}}},\\ r_{\text{ave}\_\min}=\frac{\sum_{n_{\min}}\left\|x_{\min}-x_{\text{center}\_\min}\right\|}{n_{\min}}. \end{array}$$

**Figure 3:**
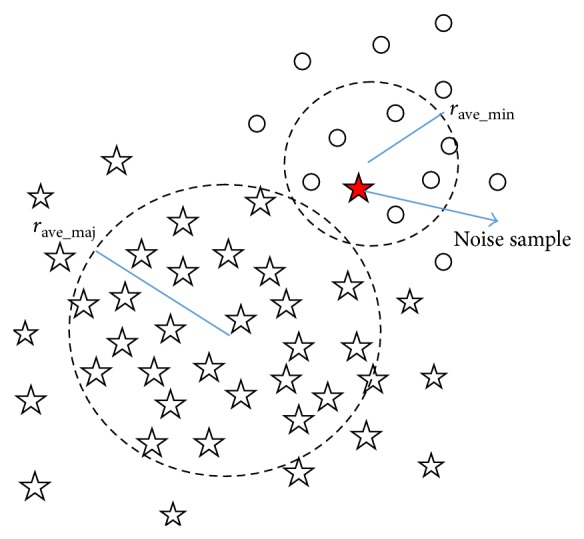
Illustration of boundary noise of majority class sample.

Let *d*_maj_ = ‖*x*_maj_ − *x*_center_maj_‖ indicate distance between a majority class sample and the center; sort *d*_maj_ of all majority class samples in an order of big to small, and take samples whose *d*_maj_ is the top 5% maximum as* Boundary Samples*. Calculate distance from Boundary Sample to the center of minority class as follows: *d*_maj_min⁡_ = ‖*x*_maj_ − *x*_center_min⁡_‖; if *d*_maj_min⁡_ < *r*_ave_min⁡_,* the Boundary Sample* is taken as* noise* which may cause the classification hyperplane move into the minority class, and they are deleted from the majority class samples. The process is illustrated in Figure 3; among them circles and pentagons denote samples of minority class and majority class, respectively, and the red solid pentagon is a detected noise sample.

After removing boundary noise of majority class samples, random undersampling processing will be executed. Our random undersampling just like dual-drawn-out in image compressing, drawing out one sample from every two-adjacent-sample, can ensure keeping the original sample distribution after undersampling and remove replicate information as well. After one time random undersampling processing, the number of majority classes will decrease a half; that is, the rate of undersampling RU = 2, and after *n* times random undersampling processing, the rate of undersampling will become RU = 2^*n*^, where *n* should be selected according to the number ratio between the two class samples.

#### 2.2.3. RU-SMOTE-SVM Classifier

Although both oversampling and undersampling algorithms can achieve the purpose of balance samples, the reserved or generated samples are not necessarily valid on the generation of decision-making interface; therefore the simple combination by one of them with SVM does not fundamentally improve the SVM classification performance for minority class.

In this research, we combine these two sampling methods for data balance and propose a SVM classification algorithm based on random undersampling and synthetic minority oversample technique (RU-SMOTE-SVM). Suppose the number ratio of the two classes samples is *N*_ratio_ = *n*_maj_/*n*_min⁡_; it needs to set the parameters *K* for synthetic new minority class sample using SMOTE method, and RU of downsampling for the majority class samples; the goal is to adjust the number of the two classes samples close to each other. In the premise of RU ≥ 2 and the range of *k* = 3~6, the two parameters of *K* and RU should be equivalent as far as possible to avoid excessive adjusting of one side. Take some examples for setting of *K* and RU. When *N*_ratio_ = 6, set RU = 2 and *K* = 3; when *N*_ratio_ = 10, set RU = 2 and *K* = 5; when *N*_ratio_ = 20, set RU = 2^2^ = 4 and *K* = 5.

The algorithm can remove noise and duplicate information of the majority of samples to improve utilization of data; in the meanwhile, it can increase the effective location of sample information in the minority class. With reserving the useful information of majority samples and making full use of minority samples, the two class samples are balanced.

**Figure 4:**
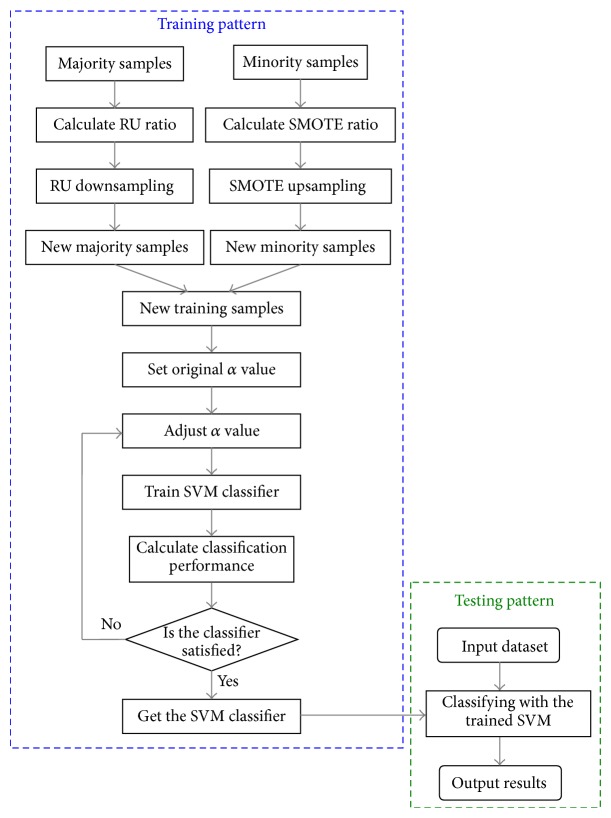
Flow chart of algorithm of RU-SMOTE-SVM classification.

The main process of our algorithm is as follows. Firstly, calculate the difference between the number of majority class and minority class samples in the training data and determine the number of removing and increasing samples, respectively. Then, reduce the majority class samples and increase the minority class samples by RU and SMOTE algorithms according to the predetermined values, respectively. Set an original value of *α*, train SVM with the new training samples, and calculate the classification parameters. Finally, adjust *α* value to get the optimum classification performance to make the classifier have better generalization ability on the unbalanced data. The training process is to solve the objective function iteratively to obtain the optimal classification hyperplane, and the ultima *α* determines the discriminate function and the rule of classification. The flow chart of our algorithm is illustrated in Figure 4.

## 3. Results and Discussions

### 3.1. Dataset

The experimental data used are low-dose CT lung images from ShengJing Hospital affiliated to Chinese Medical University, Beijing Xuanwu Hospital, and the U.S. National Cancer Institute (NCI) issued by the Lung Image Data Union (Lung Image Database Consortium, LIDC) [32]. Each scan contains a varying number of image slices. The images were captured by different CT scanners including Siemens, Toshiba, Philips, and General Electric. All images were of the size 512 × 512 pixels. The pixel size varied from 0.488 mm to 0.762 mm, and the slice thickness ranged from 1.25 mm to 3.0 mm.

**Figure 5:**
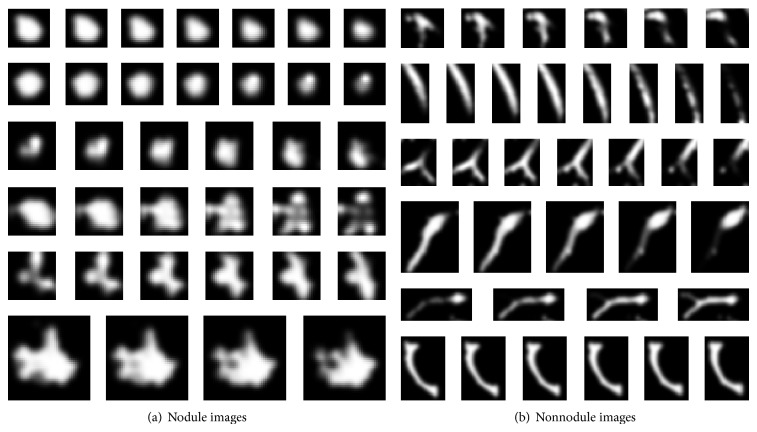
Nodule and nonnodule sequent images.

We choose 120 thoracic CT scans for the experiments. To set the dataset, we extracted nodule and nonnodule regions from the lung images, and they are all examined by expert radiologists. We created the nodule and nonnodule regions in forms of volume data, that is, *a* pixels ×  *b* pixels ×  *c* layers; *a*, *b*, and *c* stand for size of the nodule or nonnodule in *x*, *y* and, *z* direction, respectively, the range of *a* and *b* is 10~50 pixels, and the range of *c* is 5~13. We create 150 nodules and 908 nonnodules for the dataset.  Figure 5 shows 6 nodule and 6 nonnodule sequent images of the dataset, and groups (a) and (b) show nodule and nonnodule images, respectively.

The method includes training and test stages. We adopted *m* × 2 Cross Validation method. That is the original dataset is randomly divided into two parts: one including 75 nodules and 454 nonnodules was used as training samples, and the other included 75 nodules and 454 nonnodules for testing. The process is repeated 5 times; that is *m* = 5.

For each sample,** 8** features are extracted for training and classification, including* four* 2D features (*circularity*,* elongation*,* compactness*, and* moment*) and* four* 3D features (*surface-area, volume, sphericity*, and* centroid-offset*); the definitions and equations of the features are explained in Appendix A.

### 3.2. Training Data Balanced by RU-SMOTE Method

**Figure 6:**
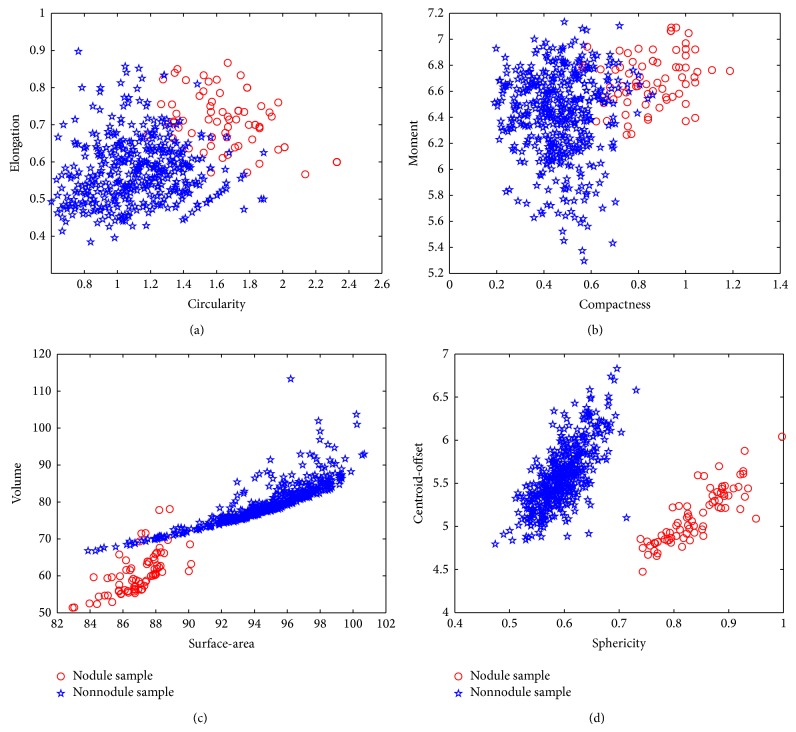
Original data distributions of 2D and 3D features. (a) Original data distribution of 2D features of* circularity* and* elongation.* (b) Original data distribution of 2D features of* compactness* and* moment.* (c) Original data distribution of 3D features of* surface-area* and* volume.* (d) Original data distribution of 3D features of* sphericity* and* centroid-offset*.

**Figure 7:**
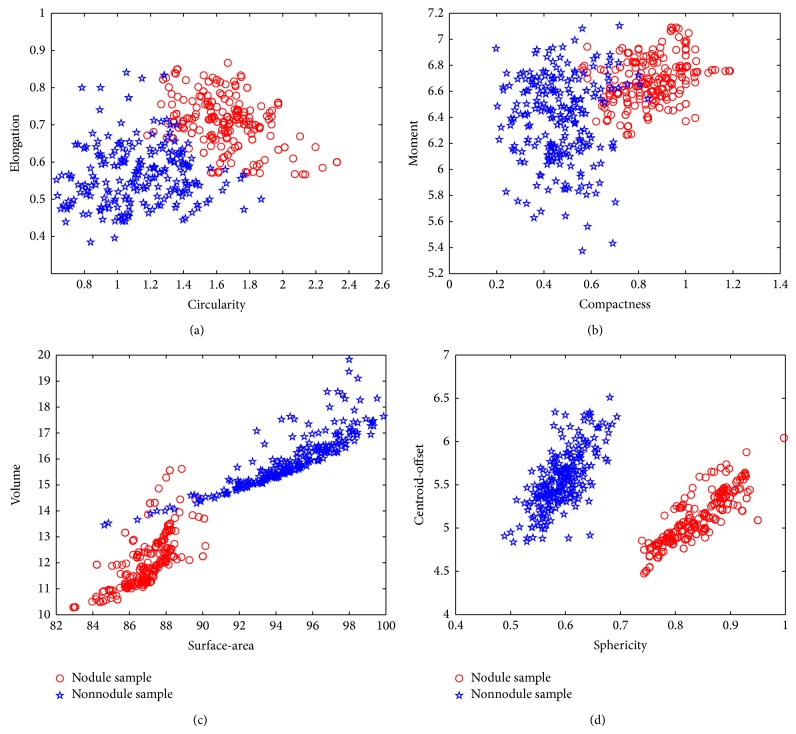
New data after balance distributions of 2D and 3D features. (a) New data distribution of 2D features of* circularity* and* elongation.* (b) New data distribution of 2D features of* compactness* and* moment*. (c) New data distribution of 3D features of* surface-area* and* volume*. (d) New data distribution of 3D features of* sphericity* and* centroid-offset*.

For every training data of 75 nodules and 454 nonnodules with** 8** features, we use RU-SMOTE method described in Section 2 to balance the samples. After the data balance, the nodule number is 225 and the nonnodule number is 227. Figures 6 and 7 give an example of the data distributions of original features and after balance, respectively.

### 3.3. Quantity Evaluation of Classification

In the classification of pulmonary nodule ROI, if nodules are judged as nonnodules and are removed directly, the nodules are not prompted by the doctor, and this will cause overlooking and misdiagnosis of nodules. Under these cases, patients tend to miss or delay the best time of treatment. However, misdiagnosis of nonnodules only increases the number of suspected cases to the doctor, and a new judgment and assessment may be given before the medical diagnosis, resulting in smaller losses. Therefore, the loss of nodules misclassification is far greater than that of nonnodules.

In view of the accuracy of rare class recognition rate which is far more important than that of the major samples, we should try to improve the recognition rate of the minority class. But the effect of majority class to accuracy standard is often greater than the minority class, resulting in the recognition rate of minority class being difficult to rise; then for unbalanced data, we need to take more attention to the minority class performance of the evaluation criteria of new classifier.

In this paper, only two classes of classification problem are taken into account, the minority is defined as positive class, and the majority is defined as negative class. Here the evaluation of confusion matrix in machine learning is introduced (as shown in Table 1).

In the confusion matrix of a two-class system, when the judgement by experts and the prediction by classifier are both positive, the result is True Positive, that is, TP; when the judgement by experts is positive, while the prediction by classifier is negative, the result is False Negative, that is, FN; when the judgement by experts is negative, while the prediction by classifier is positive, the result is False Positive, that is, FP; when the judgement by experts and the prediction by classifier are both Negative, the result is True Negative, that is, TN.

Quantitative evaluation indexes for classifier can be defined by confusion matrix as follows.

The overall classification accuracy rate is

(8) $$\begin{array}{c} \text{Accuracy}=\frac{\left(\text{TP}+\text{TN}\right)}{\left(\text{TP}+\text{TN}+\text{FP}+\text{FN}\right)}. \end{array}$$

Probability of TP is

(9) $$\begin{array}{c} \text{TPR}=\frac{\text{TP}}{\left(\text{TP}+\text{FN}\right)}. \end{array}$$

Probability of FP is

(10) $$\begin{array}{c} \text{FPR}=\frac{\text{FP}}{\left(\text{FP}+\text{TN}\right)}. \end{array}$$

Classification accuracy rate of positive class is

(11) $$\begin{array}{c} \text{ac}{\text{c}}^{+}=\frac{\text{TP}}{\left(\text{TP}+\text{FN}\right)}. \end{array}$$

Classification accuracy rate of negative class is

(12) $$\begin{array}{c} \text{ac}{\text{c}}^{-}=\frac{\text{TN}}{\left(\text{TN}+\text{FP}\right)}. \end{array}$$

A commonly used dataset of unbalanced data classification performance evaluation criteria is geometric mean of *G*-mean, which is widely used in the performance evaluation of the unbalanced data set. *G*-mean is defined as

(13) $$\begin{array}{c} G\text{-mean}=\sqrt{\text{ac}{\text{c}}^{+}\times \text{ac}{\text{c}}^{-}}. \end{array}$$

*G*-mean maintains a balance between classification accuracies of the two classes.

For the evaluation of support vector machines, a function of *F-measure* is a way of evaluation of accuracy and sensitivity of the classification results for positive class. Here the accurate rate of classification of positive class is defined as

(14) $$\begin{array}{c} P=\frac{\text{TP}}{\left(\text{TP}+\text{FP}\right)}. \end{array}$$

Sensitivity of the classification of positive class is

(15) $$\begin{array}{c} R=\frac{\text{TP}}{\left(\text{TP}+\text{FN}\right)}. \end{array}$$

The evaluation function of *F*-measure can be gotten as follows:

(16) $$\begin{array}{c} F\text{-measure}=\frac{2\times P\times R}{P+R}. \end{array}$$

Obviously, the optimum of classification is that *F*-measure gets the maximum value 1.

As described in Section 3.1, the dataset includes 150 nodule and 908 nonnodule images, and half of the original nodule and nonnodule data are randomly used for training and testing, respectively. For comparing, the training data are balanced by our RU-SMOTE method and SMOTE, respectively, so the data distribution of training and testing is as Table 2.

Classification experiments are implemented by SVM methods using the datasets as in Table 2. There are four classifiers constructed for the experiments; SVM classifier and biased-SVM classifier [23] use original training datasets; SMOTE-SVM classifier is constructed by training samples balanced by SMOTE method and SVM; RU-SMOTE-SVM classifier is constructed by training samples balanced by RU-SMOTE method and SVM. All the four classifiers use the same testing samples datasets. The parameters of the four classifiers are set as follows:

1. SVM classifier: kernel function is RBF; set *C* = 10;
2. Biased-SVM classifier: kernel function is RBF, as *N*_ratio_ = 454 : 75 ≈ 6, so set *C*_+_ = 10, *C*_−_ = round(*C*_+_/6) = 2;
3. SMOTE-SVM classifier: kernel function and parameters are set as the SVM classifier, in the SMOTE algorithm; set *N* = 5, *K* = 5;
4. RU-SMOTE-SVM classifier: kernel function and parameters are set as the above SVM classifier, set the rate of random undersampling RU = 2, and set the SMOTE parameter *N* = 3, *K* = 3.

Training and testing experiments have been done for 5 times using datasets as described in Section 3.1; the average results of the four classifiers are given in Table 3.

From Table 3, we can see that, for the same testing datasets, RU-SMOTE-SVM classifier gets the most number of TP, the highest accuracy rate, *G*-mean, and *F*-measure among the four classifiers. For ROI classification, the loss of misjudgment of nodule to nonnodule is greater than that of misjudgment of nonnodule to nodule, so the value of TP is more important than the value of FP. The higher the value of the TP is, the better the classifier is. So, RU-SMOTE-SVM classifier is with the best performance for ROI classification among the four classifiers.

### 3.4. Discussions

More experiments are carried out under different ratio between majority and minority samples in training dataset, and the influences to nodule classification performances are examined. New training datasets with different *N*_ratio_ were constructed; the distributions of datasets are shown in Table 4.

**Figure 8:**
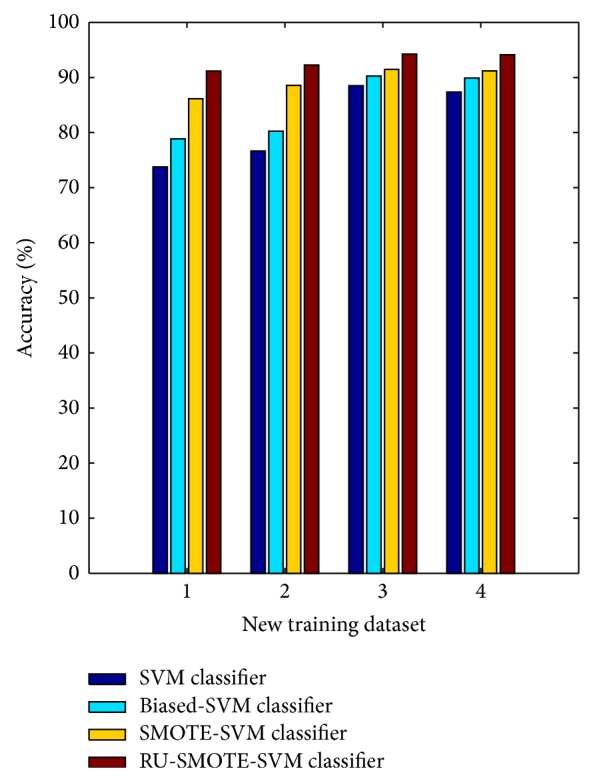
Compare of classification accuracy under new training datasets.

To compare the performance of the four classifiers under the new training datasets, the same testing dataset was used in the experiments.  Figure 8 gives the compare of accuracy for the four classifiers under the four new training datasets. We can see that RU-SMOTE-SVM classifier gets the highest classification accuracy under all the four training datasets.

The average classification accuracy of the four classifiers under different training datasets is 81.57%, 84.82%, 89.33%, and 92.94%, respectively. Different ratio between two class samples of training dataset brings the least effects upon the classification performance to RU-SMOTE-SVM classifier. On the contrary, SVM classifier and biased-SVM classifier suffer the effects of sample ratio of training dataset obviously.

## 4. Conclusions

In this paper, for the problem of unbalanced data for pulmonary ROI classification, we propose a novel SVM classifier combined with RU and SMOTE resampling technique for lung nodule detection. The combinations of the two resampling methods not only achieve balanced training samples, but also remove noise and duplicate information in the training sample and retain useful information to improve the effective of data utilization, so they improve performance of SVM algorithm for pulmonary nodules classification under the unbalanced data. Eight features including 2D and 3D features are extracted for training and classification. Experimental results show that, for different sizes of datasets, our RU-SMOTE-SVM classifier gets the highest classification accuracy among the four kinds of classifiers; the average classification accuracy is more than 92.94%. It is suitable for the application in clinical lung cancer CAD system.

## Figures and Tables

**Figure 9:**
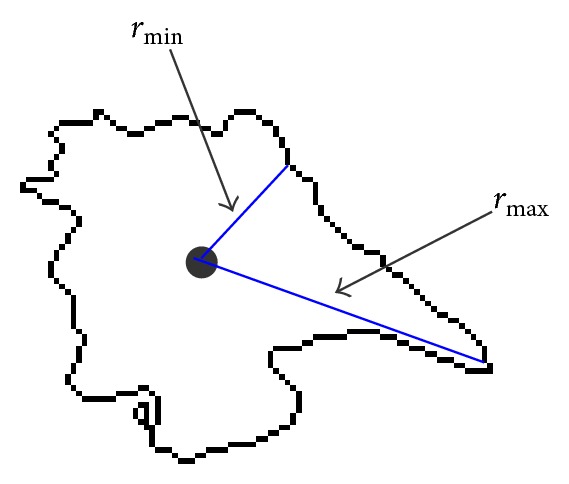
Illustration of *r*_min⁡_ and *r*_max⁡_.

**Figure 10:**
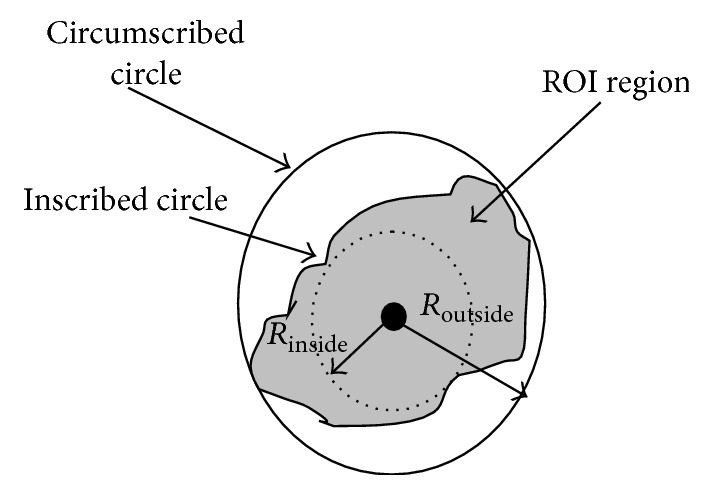
Illustration of the inscribed circle and circumscribed circle of ROI.

**Figure 11:**
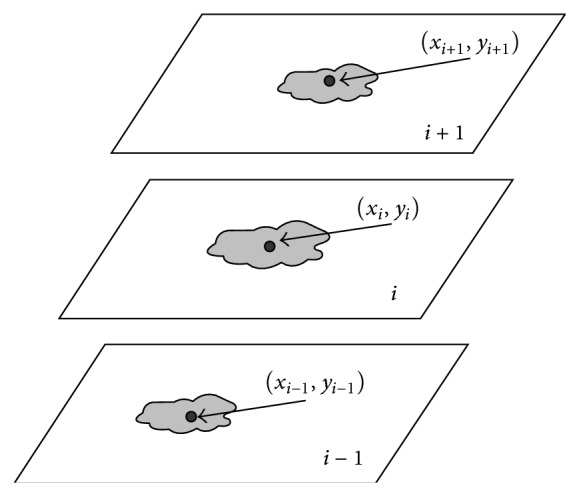
Illustration of 3D feature* centroid-offset*.

**Table 1:** Confusion matrix for two classes.

Confusion matrix	Forecasting positive by classifier	Forecasting negative by classifier
Judging positive by experts	TP	FN
Judging negative by experts	FP	TN

**Table 2:** Distribution list of ROI sample datasets.

ROI dataset	Number of nodules	Number of nonnodules
Original training samples	75	454

Balanced training samples by SMOTE method	375	454

Balanced training samples by RU-SMOTE method	225	227

Testing data	75	454

**Table 3:** The average results of the four classifiers.

Evaluation index classifier	TP	FN	FP	TN	Accuracy	*G*-mean	*F*-measure
SVM classifier	26	49	5	449	0.8979	0.5855	0.4906
Biased-SVM classifier	46	29	24	430	0.8998	0.7622	0.6344
SMOTE-SVM classifier	51	24	17	437	0.9225	0.8090	0.7133
RU-SMOTE-SVM classifier	58	17	16	438	0.9376	0.8638	0.7785

**Table 4:** Distribution list of new datasets.

	Datasets	Number of nodules	Number of nonnodules	*N*_ratio_
New training dataset	Number 1	25	454	20
	Number 2	45	454	10
	Number 3	75	150	2
	Number 4	75	300	4

Testing dataset		75	454	6

## Appendix

### A. Features Used in This Study

#### A.1. 2D Features

The ROI samples are either nodule or nonnodule image sequence; they are in forms of volume data of *a* pixels ×  *b* pixels ×  *c* layers. Here 2D features are first extracted from each layer of ROI sample. Then calculate the average of all layers.

##### A.1.1. Circularity

*Circularity* reflects the similar degree of ROI region to a circle as follows:

(A.1) $$\begin{array}{c} \text{Circularity}=4\pi \frac{\text{Area}}{L^{2}}. \end{array}$$

Here, *P* is the perimeter of ROI.

##### A.1.2. Elongation

*Elongation* measures the elongation or asymmetry degree of an object. It is calculated through (A.2), where *r*_min⁡_ is the measurement from the centroid to the nearest point on the boundary, while *r*_max⁡_ is the measurement from the centroid to the farthest point on the boundary, as illustrated in Figure 9. One has

(A.2) $$\begin{array}{c} \text{Elongation}=\frac{r_{\min}}{r_{\max}}. \end{array}$$

From (A.2), the range of* elongation* is 0~1, and the smaller the value is, the more asymmetric the ROI is.

##### A.1.3. Compactness

*Compactness* of ROI is defined as

(A.3) $$\begin{array}{c} \text{Compactness}=\frac{R_{\text{inside}}}{R_{\text{outside}}}. \end{array}$$

*R*_inside_ is the radius of inscribed circle of ROI, and *R*_outside_ is the radius of circumscribed circle of ROI, as illustrated in Figure 10. The range of* compactness* is 0~1. If the value of compactness approximates to 1, ROI is compact and closed to a circle.

##### A.1.4. Moment

*Moment* of ROI is defined as

(A.4) $$\begin{array}{c} \text{moment}=\overset{P-1}{\underset{i=0}{\sum }}\overset{\;Q-1}{\underset{\;j=0}{\sum }}\frac{{\left(f\left(i,j\right)\right)}^{2}}{1+\left|i-j\right|}. \end{array}$$

*f*(*i*, *j*) is normalized gray-value of pixels of ROI; *P* and *Q* are the number of row and column, respectively.

#### A.2. 3D Features

##### A.2.1. Surface-Area

One has

(A.5) $$\begin{array}{c} A=\underset{(x,y)\subset S_{i}}{\sum }P\left(x,y\right), \end{array}$$

where *P*(*x*, *y*) is perimeter of ROI, which is pixel number of ROI boundary, and *S*_*i*_ is the *i*th layer of ROI.

##### A.2.2. Volume

One has

(A.6) $$\begin{array}{c} V=N. \end{array}$$

Here *N* is sum of numbers of pixels whose gray scale is nonzero in all the ROI layers, and it is defined as the volume of three-dimensional ROI.

##### A.2.3. Sphericity

One has

(A.7) $$\begin{array}{c} \text{sphericity}=\frac{6\sqrt{\pi }V}{A^{3/2}}, \end{array}$$

where *V* is the* volume* and *A* is the surface-area of the ROI region.* Sphericity* measures how much the shape of the object approximates to a spherical shape.

##### A.2.4. Centroid-Offset

Consider

(A.8) $$\begin{array}{c} \text{centroid-offset}=\overset{K}{\underset{i=1}{\sum }}\left(\left|x_{i}-\overset{-}{x}\right|+\left|y_{i}-\overset{-}{y}\right|\right), \end{array}$$

where *K* is the number of ROI layers, (*x*_*i*_, *y*_*i*_) is the coordinate of the centroid of the *i*th layer, and $\left(\overset{-}{x},\overset{-}{y}\right)$ is the average coordinate of all the ROI layers. It is illustrated in Figure 11.
